# The Ebola Virus: From Basic Research to a Global Health Crisis

**DOI:** 10.1371/journal.ppat.1005093

**Published:** 2015-08-13

**Authors:** Robert V. Stahelin

**Affiliations:** 1 Department of Biochemistry and Molecular Biology, Indiana University School of Medicine-South Bend, South Bend, Indiana, United States of America; 2 Department of Chemistry and Biochemistry, the Eck Institute for Global Health, and the Boler-Parseghian Center for Rare and Neglected Diseases, University of Notre Dame, Notre Dame, Indiana, United States of America

For me, interest in Ebola virus (EBOV) started in the late ‘90s, as an undergraduate biochemistry major on a fishing trip with the book *The Hot Zone* in hand. Richard Preston’s ability to communicate how EBOV ravages the human body and yet could be lurking in an animal reservoir was both frightening and thought-provoking. I went on to graduate school, decided to pursue a PhD in lipid–protein interactions, and stayed clear of viruses for the time being. My postdoctoral training then centered on lipid–protein interactions in live human cells and mostly focused on proteins involved in cancer signaling.

When I started my own lab in 2006, I had a niche in biochemistry and biophysics of lipid–protein interactions and was particularly interested in discovery of new lipid-binding proteins. I once again picked up my reading of virology and realized my tools and expertise could be utilized to study how viruses replicated in human cells. In order to sustain the infection and be transmitted from a person exhibiting symptoms, EBOV needs to enter, replicate, and exit a human cell. This is an impressive cycle given that EBOV only harbors seven genes in its genome compared to approximately 4,000 in some bacteria and approximately 19,000 in humans. How the virus moves through this cycle was the question that drove my interest in studying EBOV.

The EBOV replication cycle had been shown to finalize with the exit from the human cell plasma membrane, the outer membrane being so important for human cell shape and stability. This meant the virus stole its lipid coat from the human cell it infected, and this fact could be a means of understanding how the virus replicates, perhaps a process that could be targeted with new therapeutics. However, EBOV is such a dangerous virus, its work is restricted to high-containment (biosafety level 4) facilities, thus limiting my research options. Fortunately, more than a decade ago, several labs discovered that just one of the seven EBOV proteins, VP40 (viral protein 40), is able to form virus-like particles (VLPs) when expressed in human cells. These VLPs are nearly indistinguishable from authentic virions and have served as an appropriate model of understanding EBOV exit from the plasma membrane. This model led us to several fundamental discoveries of how the EBOV VP40 protein alone interacts with negatively charged lipids in human cells, induces filamentous alterations in membrane shape, and ultimately mediates the pinching-off of the human cell membrane to form a new EBOV particle.

The EBOV lipid coat also provides the virus a way to enter human cells, as EBOV mimics damaged cells that expose the lipid phosphatidylserine (PS) on their outer coat. Damaged cells exposing PS are normally cleared by the immune system when human cells take up viruses, thereby exposing PS on their outer coat. The presence of the viral genome means the virus can then replicate in cells that have engulfed the virus. In essence, the virus is using our own lipids against us.

Failure to recognize the role of basic science in studying EBOV and other viruses may endanger the future enterprise and training of researchers interested in these pathogens. Admittedly, each project on EBOV doesn’t lead to a new drug, vaccine, or diagnostic test, but overall our basic approaches have led to drug screening assays against the EBOV human cell exit pathway. Additionally, viruses are great tools to study and understand human cell biology, since viruses will often encode their proteins to hijack human cell pathways. These models should transform our understanding of how human cells work, such as movement and trafficking of membranes, overcoming energetic barriers to change membrane shape, and mediating a multitude of signaling events.

The current Ebola virus (EBOV) outbreak in western Africa has caused more than 26,000 infections and has claimed at least 11,000 lives as of June 15, 2015. This is by far the largest outbreak of the virus in history, but despite some promising drugs, antibodies, and vaccines being developed, we still lack much fundamental knowledge of how this virus replicates inside human cells. Future basic science funding could help to better understand how EBOV spreads from cell to cell and why some patients survive and others don’t, and it could provide more rapid diagnostic assays for field analyses.

**Image 1:**
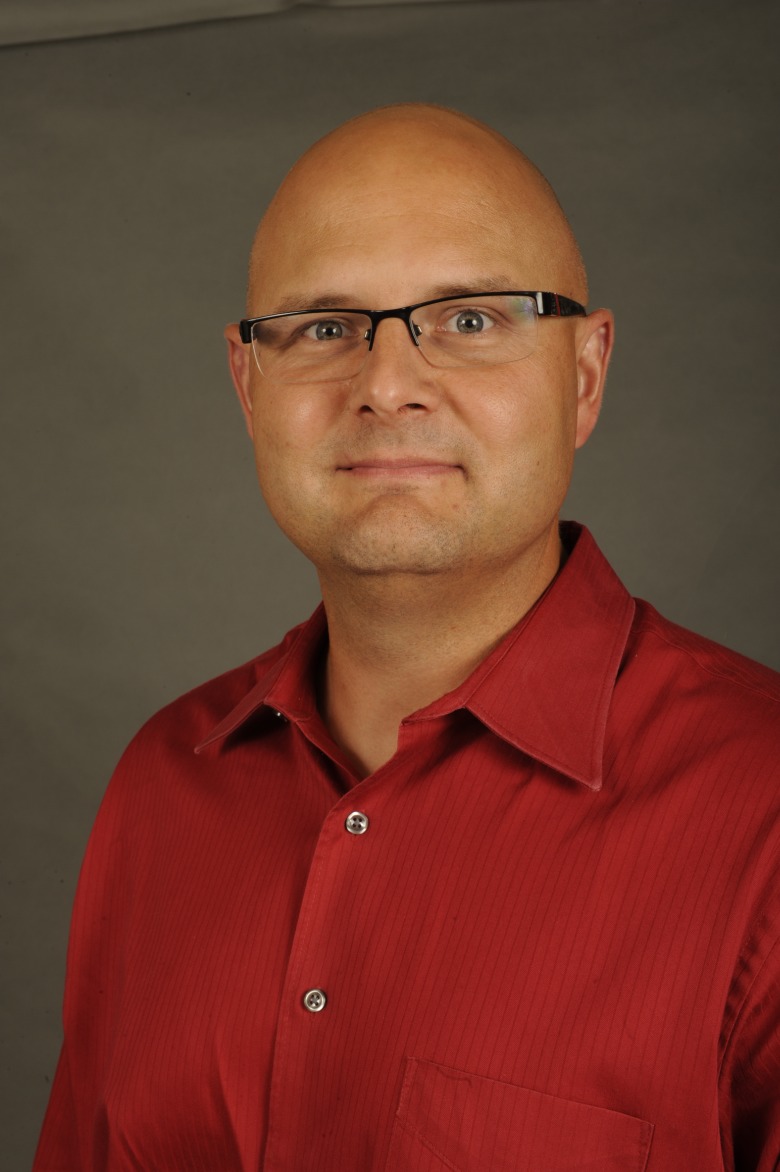
Robert V. Stahelin.

